# Anatomic and Functional Outcomes of Scleral Buckling Surgery for Advanced Retinopathy of Prematurity: A Systematic Review and Proportional Meta-Analysis

**DOI:** 10.7759/cureus.76000

**Published:** 2024-12-19

**Authors:** Eleni Kottaridou, Aikaterini K Seliniotaki, Asimina Mataftsi, Anna-Bettina Haidich, Maria Lithoxopoulou, Anna Koutra, Christos G Dragonas

**Affiliations:** 1 Department of Accident and Emergency, St Thomas' Hospital, Guy's and St Thomas' NHS Foundation Trust, London, GBR; 2 2nd Department of Ophthalmology, School of Medicine, Faculty of Health Sciences, Aristotle University of Thessaloniki, Thessaloniki, GRC; 3 Department of Hygiene, Social-Preventive Medicine and Medical Statistics, School of Medicine, Faculty of Health Sciences, Aristotle University of Thessaloniki, Thessaloniki, GRC; 4 Neonatal Intensive Care Unit, 2nd Department of Neonatology, School of Medicine, Faculty of Health Sciences, Aristotle University of Thessaloniki, “Papageorgiou” General Hospital of Thessaloniki, Thessaloniki, GRC; 5 Faculty of Medicine, Aristotle University of Thessaloniki, Thessaloniki, GRC; 6 Department of Trauma and Orthopaedics, Barts Health NHS Trust, London, GBR

**Keywords:** paediatric ophthalmology, retinal detachment (rd), retinopathy of prematurity, retinopathy of prematurity (rop), scleral buckle, scleral buckling, vitreoretinal surgery

## Abstract

A significant percentage of patients with retinopathy of prematurity (ROP) who progressed to stages 4 and 5 of ROP will require surgical intervention. Scleral buckling surgery is widely employed for the restoration of retinal detachment in advanced cases of ROP.

This systematic review and meta-analysis aim to review the anatomical and visual outcomes following scleral buckling surgery in ROP of stages 4 and 5.

We performed a systematic literature search in two databases (MEDLINE and EMBASE), according to the Preferred Reporting Items for Systematic reviews and Meta-Analyses (PRISMA) guidelines, from January 2000 to July 2024 for studies examining the anatomical and functional outcomes resulting from the scleral buckling procedure in premature infants with advanced ROP. We used the inverse variance random effects model to calculate pooled effect estimates and conducted a single-arm proportional meta-analysis. The study was conducted based on a prespecified protocol registered in the International Prospective Register of Systematic Reviews (PROSPERO) database with registration number CRD42024557314.

Ten eligible studies for our systematic review were identified. The pooled prevalence for the anatomic outcome, that is the proportion of retinal reattachment, was estimated at 65% (95% Cl [0.49 - 0.82], p-value 0 < 0.0001). The visual outcome was investigated through five studies. The prevalence of normal visual acuity or mild visual impairment, defined as visual acuity between 6/18 and 6/60 as per the World Health Organisation (WHO) visual impairment recommendations, was calculated at 41% (95% Cl [0.29; 0.52], p-value < 0.0001, I^2^ =0.02%). The follow-up period ranged between one month and 88 months across all studies.

Scleral buckling surgery contributes to the mitigation of the progression of retinal detachment in premature infants afflicted with advanced ROP. The anatomical outcomes demonstrate more favourable results in comparison to visual outcomes. Future prospective studies with extended and explicit follow-up periods and larger populations would significantly enhance the existing body of knowledge in this field.

## Introduction and background

Retinopathy of prematurity (ROP) is a vasoproliferative disorder that affects premature infants and low-birth-weight (BW) infants. According to the World Health Organisation (WHO), it is a leading cause of visual impairment and blindness in children worldwide [1], with 25% of all extremely preterm babies being affected and moderately preterm babies with poorly monitored oxygen therapy being at higher risk [2,3]. In 2010, Blencowe et al. [4] demonstrated that approximately 20,000 preterm babies, out of 184,700 infants with any stage of ROP, became blind or severely visually impaired because of the condition.

It is closely linked to low BW and gestational age (GA), exhibiting an inversely proportional correlation, which is used as the basis of screening guidelines for ROP globally, including in the USA [5].

Previous literature has indicated three epidemics of ROP, starting in the 1940s-1950s in industrialised countries due to unmonitored oxygen supplementation, leading to the second epidemic in the 1970s with the development of advanced neonatal care and the survival of extremely premature infants and finally concluding in the third epidemic in the 1990s, which was expressed in low- and middle-income countries, extending from Eastern Europe and Latin America to the East and South Asia, due to a combination of optimised care, increased survival rates of premature infants, and varying levels of oxygen regulation [6]. Recent research indicates a continuous increase in the incidence of ROP in the United States, indicating the risk of a potential upcoming ROP outbreak. The epidemiology cohort study by Bhatnagar et al. attributes this rise in ROP cases to enhanced screening protocols, along with existing racial and ethnic disparities in the healthcare system that result in limited access to care and higher healthcare expenses [7]. These findings prove that ROP remains an important public health problem with global implications.

Several studies have investigated the treatment options for different stages of ROP, as recommended by the International Classification of Retinopathy of Prematurity (ICROP). The Multicentre Trial of Cryotherapy for Retinopathy of Prematurity (CRYO-ROP) study in 1986 established the threshold disease (defined as five contiguous or eight cumulative clock hours of stage 3 ROP in zone 1 or zone 2 with plus disease) and the use of cryotherapy to overcome the disease progression. Subsequently, the Early Treatment for Retinopathy of Prematurity (ETROP) study in 1999 introduced laser photocoagulation for pre-threshold disease, whereas succeeding studies, such as the Bevacizumab Eliminates the Angiogenic Threat of Retinopathy of Prematurity (BEATROP) study in 2008, the RAINBOW in 2016, and the FIREFLEYE and BUTTERFLEYE studies in 2019, explored the treatment with anti-vascular endothelial growth factor (anti-VEGF) agents and their optimal dosage in ROP [8-13].

According to ICROP, ROP is classified into three zones by its location, five stages by its severity and three categories by its vascular features in the posterior pole. More specifically, stage 4 ROP describes the partial detachment, either exudative or tractional, whereas stage 5 ROP designates the total retinal detachment [14]. Various surgical methods have been employed in the restoration of retinal detachments linked to ROP, including both vitrectomy and scleral buckling surgeries. Comparative studies with a limited population have proved the predominance of vitrectomy against scleral buckling surgery regarding the percentage of reattachment as well as the improvement of the visual status of patients with advanced ROP (Stage 4, Stage 5) [15,16]. Given the high level of expertise required in paediatric vitrectomy, as well as the limited availability of specialized equipment in neonatal intensive care units (NICUs), it would be worthwhile to investigate the outcomes that occurred by scleral buckling surgery [17]. This systematic review and meta-analysis aim to assess the anatomical outcome, defined as the success of retinal reattachment, and the visual outcome, which represents the improvement of visual acuity (VA), following scleral buckling surgery in patients with advanced ROP.

## Review

Methods

We present a systematic review and single-arm meta-analysis as recommended by the Preferred Reporting Items for Systematic reviews and Meta-Analyses (PRISMA) 2024 Statement. The study was conducted based on a prespecified protocol registered in the International Prospective Register of Systematic Reviews (PROSPERO) database with registration number CRD42024557314.

Eligibility Criteria

The protocol for this study was based on a modified variation of the PICO (Population, Intervention, Control, Outcomes) model, adjusted for observational studies. We included observational studies that assessed the anatomical and visual outcomes following scleral buckling surgery in patients with advanced ROP. Eligible studies included preterm infants born 37 weeks of postmenstrual age or younger with stage 4 or 5 ROP who received scleral buckling surgery as a primary surgical intervention regardless of previous additional treatments, such as cryotherapy, laser photocoagulation or anti-VEGF injections. Exclusion criteria were: 1) Case reports, literature reviews, letters to editors, and animal studies, 2) Studies published prior to 2000, 3) Patients with vitreoretinal conditions other than ROP, 4) Patients with early stages of ROP who developed retinal detachment caused by other factors, such as trauma, 5) Primary surgical intervention other than scleral buckle (such as vitrectomy) and 6) Studies published in a language other than English. 

Search Strategy and Study Selection

A literature search was performed on MEDLINE (via PubMed) and EMBASE (via Ovid) for studies that were published from January 2000 to July 2024. The search strategy employed encompassed Medical Subject Headings (MeSH) and free-text terms that delineate the exposure variable as well as the population of interest, as indicated in Table 1. Two investigators (EEKK, AKS) independently screened the existing literature and assessed the eligibility of individual studies. Duplicated studies were identified and removed. All studies were first evaluated by their titles and abstracts and subsequently by their full texts based on prespecified criteria. Consensus was achieved following deliberations regarding the conflicts between the two reviewers.

**Table 1:** Search strategies for all databases

Database	Search strategy
MEDLINE (via PubMed)	(((((((retinopathy of prematurity) OR (retrolental fibroplasia)) OR (rop)) OR (retinopathy of prematurity[MeSH Terms])) OR (fibroplasias, retrolental[MeSH Terms])) OR (fibroplasia, retrolental[MeSH Terms])) OR (retrolental fibroplasia[MeSH Terms])) AND (((((scleral buckling[MeSH Terms]) OR (bucklings, scleral[MeSH Terms])) OR (scleral buckling)) OR (scleral buckle)) OR (scleral buckl*)), 2000-2024
EMBASE (via Ovid)	#1 (retinopathy of prematurity.mp.) OR (retrolental fibroplasia/) in Article title, Abstract, Keywords #2 (scleral buckle.mp.) OR (sclera buckling procedure/) OR (scleral buckle/) in Article title, Abstract, Keywords Combined: #1 AND #2, 2000-2024

Data Extraction

Two reviewers (EEKK, CGD) conducted data extraction independently utilising a predesigned form, which included the following variables: 1) First author’s name, 2) Publication year, 3) Study design, 4) Sample size measured in the number of eyes, 5) Gestational age measured in weeks, 6) Birth weight measured in grams, 7) Follow-up duration measured in weeks, 8) Anatomical outcome and 9) Visual outcome. The anatomical outcome was defined as the number of patients with total reattachment following scleral buckling surgery and it was expressed in both the number of events and percentages. A scale of categories for visual impairment, as recommended by the WHO, based on the codes for blindness and low vision in the 10th revision of the International Classification of Diseases (ICD-10), was used for the evaluation of the visual outcome after scleral buckling surgery, as indicated in Table 2 [18,19]. More specifically, six categories were delineated: Category 1 for patients with VA from 6/18 (or 3/10 or 20/70) to 6/60 (or 1/10 or 20/200), Category 2 for VA from 6/60 (1/10 or 20/200) to 3/60 (or 1/20 or 20/400), category 3 for VA from 3/60 (or 1/20 or 20/400) to 1/60 (finger - counting at 1 metre or 1/50 or 5/300), Category 4 for VA from 1/60 (finger - counting at 1 metre or 1/50 or 5/300) to light perception, Category 5 for patients with no light perception and Category 9 for patients with undetermined, specified or missing results. A previously described technique modified from Zipf [20] was used in cases of VA measurements other than Snellen VA tests, including fixation patterns [21-23]. The outcome was recorded as the number of patients with the event and was also expressed in percentages. In studies comparing scleral buckling surgery and vitrectomy, all extracted data referred to those with singular scleral buckling surgery. Anatomical and visual outcomes were classified as successful if no subsequent surgeries were required. Patients who received subsequent scleral buckle surgery or vitrectomy were classified as unsuccessful events.

**Table 2:** Categories of visual impairment as per the WHO Data from [18,19]

Category of visual impairment	Visual acuity with best possible correction	
	Maximum less than:	Maximum equal to or better than:
1	6/18 3/10 (0.3) 20/70	6/60 1/10 (0.1) 20/200
2	6/60 1/10 (0.1) 20/200	3/60 1/20 (0.05) 20/400
3	3/60 1/20 (0.05) 20/400	1/60 (finger-counting at 1 metre) 1/50 (0.02) 5/300 (20/1200)
4	1/60 (finger-counting at 1 metre) 1/50 (0.02) 5/300	Light perception
5	No light perception	
9	Undetermined or unspecified	

Risk of Bias Assessment

The studies were evaluated for biases in accordance with the Joanna Briggs Institute (JBI) Critical appraisal tool for case studies and cohort studies [24]. We decided to utilise the JBI critical appraisal tool, as it is the sole instrument recommended for case series studies. Consequently, all included observational studies were assessed using the same tool, irrespective of the study type, thereby enhancing the reliability of our findings. The evaluation of individual studies was conducted by two researchers (EEKK, CGD) independently. Any discrepancies that arose were resolved through discussion.

Data Synthesis and Analysis

We conducted a proportional meta-analysis using the R software for statistical computing and graphics (R Foundation for Statistical Computing, Vienna, Austria), version 4.1.1. and the meta R package.

We postulated the existence of clinical differences and variations, thus employing the random effects model. Both of our outcomes were dichotomous and categorical, for which we utilised the inverse variance method to analyse the data. The primary outcome delineated the success of retinal attachment after scleral buckling surgery in advanced ROP. For the visual outcome, we assumed that Category 1 patients are classified as “success,” whereas Categories 2, 3, 4, and 5 patients are categorized as “failure” regarding the restoration of the VA. Category 9 patients were omitted in the meta-analysis as they represent missing and unspecified data. After calculating the individual proportions for each variable, we conducted a pooled proportional analysis of the anatomical and visual outcomes. The results were graphically presented in a forest plot, measuring the confidence interval (Cl) and p-value with a significance level established to 0.05 for all analyses.

Results

Review of Studies

**Figure 1:**
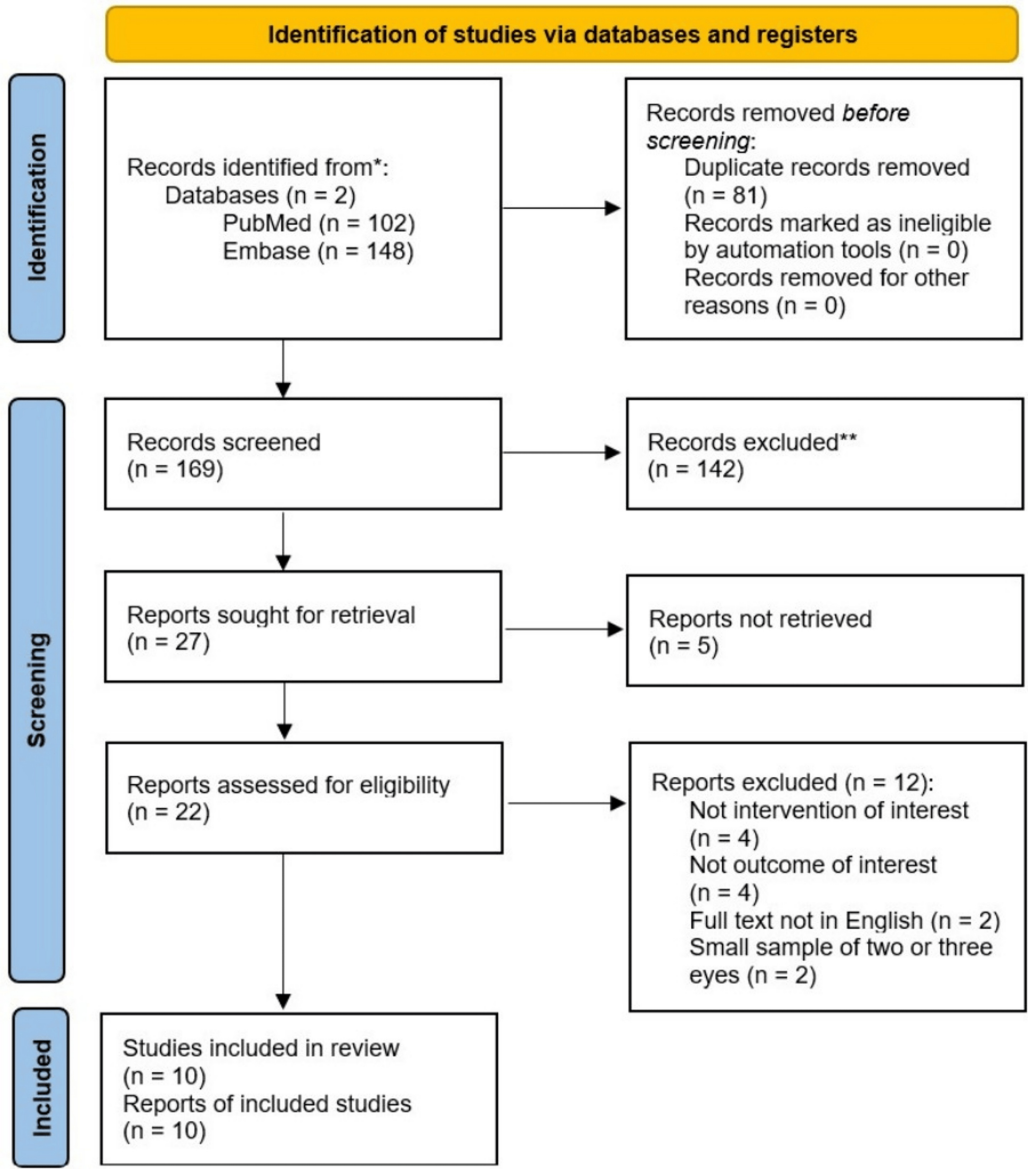
Preferred Reporting Items for Systematic Reviews and Meta-Analyses (PRISMA) 2020 flow diagram

Study selection: Our literature search yielded a total of 250 studies from two databases, namely, MEDLINE (PubMed) and EMBASE. A total of 169 records were screened based on their titles and abstracts, following the removal of 81 duplicate studies. In five studies, the full text was not accessible. Of the final 22 studies that were assessed for their eligibility, eight studies were excluded due to discrepancies in the formulation of the research question, including those that investigated the outcomes of scleral buckling surgery following vitrectomy. Additionally, non-English studies were excluded from the systematic review. Ultimately, two studies were excluded due to small sample sizes, having assessed 2 or 3 eyes in total, as part of a larger study. This study was excluded to prevent duplication of the population. Consequently, 10 studies were deemed eligible for inclusion in the systematic review [15,17,25-32], all of which were incorporated into the proportional single-arm meta-analysis. The screening process is summarised in the PRISMA flowchart in Figure 1.

Study characteristics: All the eligible studies were observational studies. In total, 214 eyes of 163 premature infants with advanced ROP were investigated in the systematic review and analysis. The mean BW of the premature infants was 874.05 grams, whereas the mean gestational age was 26.02 weeks. Only in one study [26], these characteristics were not available. The mean postmenstrual age at the time of surgery was 42.482 weeks. Data for the mean postmenstrual age were available in only five studies [15,17,27,28,31]. Of the 214 eyes, 148 eyes were diagnosed with stage 4A ROP, 58 eyes with stage 4B ROP and seven eyes with stage 5 ROP. Only one eye was not classified within a specific stage; however, it was characterized as advanced ROP, that is, ROP of stage 4 plus instead. The postoperative follow-up period was measured differently across various studies, ranging from weeks to months. The mean follow-up was 28.3 months.

Different types of preoperative or concurrent nonsurgical interventions were applied to patients across different studies. We calculated that 40.19% of all eyes sustained prior laser photocoagulation therapy, 4.2% of those had prior cryotherapy and 10.28% of all eyes received anti-VEGF treatment before the scleral buckling surgery. On the contrary, 7.48%, 5.14% and 12.62% of all eyes received concurrent laser photocoagulation, cryotherapy and anti-VEGF treatment, respectively.

In all studies, the scleral buckling surgery was used to treat the retinal detachment, which occurred as a progress of advanced ROP of stages 4A, 4B and 5. More notably, Hartnett et al. [30] included patients with retinal detachment, detached macula and vitreous haemorrhage who either received vitrectomy or scleral buckling. However, only patients who were treated with scleral buckling were included in our study. All surgical procedures were performed by experienced vitreoretinal surgeons. The surgical procedure was described in detail in most studies [17,25,27,28,31]. In cases of encircling scleral buckle, a #40 or #41 encircling band was used for the encircling procedure that was sutured at the position of the traction by the proliferative membranes or behind the rectus muscle insertions. Papageorgiou et al [17] specifically placed a silicone #240 band circumferentially as close to the ridge as possible which was sutured across all four quadrants. A similar technique was also followed by Shah et al [25]. During segmental scleral buckling surgery, a #506 buckle was used for the encircling procedure, as described by Futamura et al [27]. Anterior chamber paracentesis was performed in some cases [17,29], whereas subretinal fluid was drained in the presence of sufficient subretinal fluid [15]. The timing of buckle removal was variable, depending on the success of the scleral buckle surgery.

The anatomical outcome was defined as the success of retinal reattachment following scleral buckling surgery in patients with advanced ROP. We calculated that in 70.56% of all eyes that were included in the systematic review, retinal reattachment was achieved after the scleral buckle. The visual outcome was determined in five studies using different scales and tools [17,26,28,29,32]. More specifically, Ratanasukon et al. [28] measured the grating acuity with Teller acuity cards and converted it to the Snellen chart equivalent to express the VA of the patients. Other studies expressed the visual outcome following scleral buckling by using gratings related to fix and follow techniques [17,29]. The results were subsequently converted to a Snellen chart equivalent, through a modified technique from Zipf [20], to prevent discrepancies between studies and to facilitate statistical agreement for meta-analytic purposes. Finally, Repka et al. [26,32] evaluated the visual outcomes as recommended by Dobson et al. [33] through a behavioural measurement of grating acuity pattern, known as the acuity card procedure. The results were categorised as favourable, below normal, poor or low vision, based on their Snellen chart equivalent, as well as light perception or absence of light perception. The remaining studies [15,25,27,30,31] were classified as Category 9, as the visual outcomes were unspecified, and the data were unavailable. All the results were later classified into six categories in accordance with the WHO classification. Of the total of 214 eyes, 11.21% achieved a visual outcome of moderate visual impairment following the scleral buckle procedure, whereas 12.15% progressed to severe visual impairment or even blindness. Specifically, 3.27% of all eyes were classified as category 2 according to the WHO, resulting in a diagnosis of severe visual impairment for the patients. Additionally, 8.79% of all eyes belonged to categories 3, 4 and 5. These patients were classified as blind. The characteristics of the included studies are described in Tables 3, 4.

**Table 3:** Study characteristics ROP: Retinopathy of prematurity

Author and year	Number of eyes	Mean birth weight in grams (range)	Mean gestational age in weeks (range)	Mean postmenstrual age in weeks (range)	Follow-up duration	ROP stage -> Number of eyes
Beyrau et al. 2003 [29]	20	718 (568-879)	25 (23-26)	-	3.5 - 28 months	4A --> 15 , 4B --> 5, 5 --> 0
Hartnett et al. 2003 [30]	19	810.4 (480-2,380)	24.16 (23-26.5)	-	20.63 (5.5 - 78 months)	4A --> 4, 4B --> 12, 5 --> 3
Hartnett et al. 2004 [15]	16	689.75 (480-780)	24.22 (23-26.5)	39.31 (35-48)	1 month	4A --> ,4 4B --> 12, 5 --> 0
Ratanasukon et al. 2006 [28]	16	1009.5 (500-1600)	28 (24-36)	39.6 (32-49)	17.3 (3 - 44 months)	4A --> 8, 4B --> 8, 5 --> 0
Repka et al. 2006 [32]	10	725	25.2	-	9 months	4A --> 6, 4B --> 2, 5 --> 2
Repka et al. 2011 [26]	9	-	-	-	6 years	4A --> 6, 4B --> 2, 5 --> 0
Fatamura et al. 2015 [27]	42	843 (494-2130)	25.3 (22-31)	41.6 (34-64)	49 (6-88 months)	4A --> 29, 4B --> 13, 5 --> 0
Shah et al. 2016 [25]	13	760 (610-1,228)	25.3 (23-30)	-	-	4A --> 9, 4B --> 2, 5 --> 2
Zhong et al. 2022 [31]	62	1,335.8 (840-2,300)	29.5 (26.6-33)	50.7 (38.7-93.3)	3.64 years (1-10.48)	4A --> 62, 4B --> 0, 5 --> 0
Papageorgiou et al. 2022 [17]	7	975 (530-1275)	27.5 (24-30)	41.2 (37.6-44)	26.4 (20-34 months)	4A --> 5, 4B --> 2, 5 --> 0

**Table 4:** Study characteristics: additional noninvasive treatment Anti-VEGF: Anti-vascular endothelial growth factor

Author and year	Laser	Cryotherapy	Anti-VEGF
Beyrau et al. 2003 [29]	Prior -> 0, Concurrent -> 0	Prior -> 0, Concurrent -> 0	Prior -> 0, Concurrent -> 0
Hartnett et al. 2003 [30]	Prior -> 18, Concurrent -> 0	Prior -> 2, Concurrent -> 0	Prior -> 0, Concurrent -> 0
Hartnett et al. 2004 [15]	Prior -> 16, Concurrent -> 0	Prior -> 0, Concurrent -> 0	Prior -> 0, Concurrent -> 0
Ratanasukon et al. 2006 [28]	Prior -> 4, Concurrent -> 5	Prior -> 7, Concurrent -> 2	Prior -> 0, Concurrent -> 0
Repka et al. 2006 [32]	Prior -> 10, Concurrent -> 0	Prior -> 0, Concurrent -> 0	Prior -> 0, Concurrent -> 0
Repka et al. 2011 [26]	Prior -> 9, Concurrent -> 0	Prior -> 0, Concurrent -> 0	Prior -> 0, Concurrent -> 0
Fatamura et al. 2015 [27]	Prior -> 0, Concurrent -> 7	Prior -> 0, Concurrent -> 0	Prior -> 0, Concurrent -> 8
Shah et al. 2016 [25]	Prior -> 12, Concurrent -> 1	Prior -> 0, Concurrent -> 0	Prior -> 0, Concurrent -> 13
Zhong et al. 2022 [31]	Prior -> 11, Concurrent -> 3	Prior -> 0, Concurrent -> 9	Prior -> 21, Concurrent -> 6
Papageorgiou et al. 2022 [17]	Prior -> 6, Concurrent -> 0	Prior -> 0, Concurrent -> 0	Prior -> 1, Concurrent -> 0

Risk of Bias

The JBI critical appraisal tool was used for the individual studies. The JBI for case series assesses the reliability and relevance of the study based on 10 questions, whereas the JBI tool for cohort studies includes 11 questions. Most of the studies adhere to methodological standards, being classified as studies of moderate [26,28,29,31,32] or high [15,17,27,30] quality, with only minimal insufficiencies or limitations. However, the study by Shah et al. [25] scored 3 out of 10 on the JBI critical appraisal tool, indicating the low quality of the study and its serious methodological flaws. These findings were interpreted with caution. The results are presented in Tables 5, 6.

**Table 5:** Risk of bias assessment using the Joanna Briggs Institute (JBI) critical appraisal tool for case series studies 1: Were there clear criteria for inclusion in the case series?, 2: Was the condition measured in a standard, reliable way for all participants included in the case series?, 3: Were valid methods used for identification of the condition for all participants included in the case series?, 4: Did the case series have consecutive inclusion of participants?, 5: Did the case series have complete inclusion of participants?, 6: Was there clear reporting of the demographics of the participants in the study?, 7: Was there clear reporting of clinical information of the participants?, 8: Were the outcomes or follow up results of cases clearly reported?, 9: Was there clear reporting of the presenting site(s)/clinic(s) demographic information?, 10: Was statistical analysis appropriate?

Author	Year	Type of study	1	2	3	4	5	6	7	8	9	10	Overall score	Overall appraisal
Beyrau et al. [29]	2003	Case series	Yes	Yes	No	Yes	Yes	No	Yes	Yes	No	Yes	7/10	Moderate quality
Ratanasukon et al. [28]	2006	Case series	Yes	Unclear	Yes	Yes	Unclear	Yes	No	Yes	Yes	Yes	7/10	Moderate quality
Shah et al. [25]	2016	Case series	Yes	Unclear	No	Yes	No	No	No	No	No	Yes	3/10	Low quality
Zhong et al. [31]	2022	Case series	Yes	Unclear	Yes	Yes	Unclear	Yes	No	Yes	No	Yes	6/10	Moderate quality
Papageorgiou et al. [17]	2022	Case series	Yes	Yes	Yes	Yes	Unclear	Yes	Yes	Yes	Yes	Yes	9/10	High quality

**Table 6:** Risk of bias assessment using the Joanna Briggs Institute (JBI) critical appraisal tool for cohort studies. 1: Were the two groups similar and recruited from the same population?, 2: Were the exposures measured similarly to assign people to both exposed and unexposed groups?, 3: Was the exposure measured in a valid and reliable way?, 4: Were confounding factors identified?, 5: Were strategies to deal with confounding factors stated?, 6: Were the groups/participants free of the outcome at the start of the study (or at the moment of exposure)?, 7: Were the outcomes measured in a valid and reliable way?, 8: Was the follow up time reported and sufficient to be long enough for outcomes to occur?, 9: Was follow up complete, and if not, were the reasons to loss to follow up described and explored?, 10: Were strategies to address incomplete follow up utilized?, 11: Was appropriate statistical analysis used?

Author	Year	Type of study	1	2	3	4	5	6	7	8	9	10	11	Overall score	Overall appraisal
Hartnett et al. [30]	2003	Retrospective Cohort	No	Yes	No	Yes	Yes	Yes	Yes	Yes	Yes	Yes	Yes	9/11	High quality
Hartnett et al. [15]	2004	Retrospective Cohort	Yes	Yes	Yes	Yes	Yes	Yes	Yes	Yes	Unclear	Unclear	Yes	9/11	High quality
Repka et al. [32]	2006	Retrospective Cohort	No	No	Yes	Yes	No	Yes	Yes	Yes	Yes	Yes	Yes	8/11	Moderate quality
Repka et al. [26]	2011	Retrospective Cohort	No	No	Yes	Yes	No	Yes	Yes	Yes	Yes	Yes	Yes	8/11	Moderate quality
Fatamura et al. [27]	2015	Retrospective Cohort	Yes	Yes	Yes	Yes	Yes	Yes	Yes	Yes	Unclear	Unclear	Yes	9/11	High quality

Meta-Analysis

We conducted a proportional single-arm meta-analysis of dichotomous data for both our primary outcomes. The anatomical outcome was expressed as the success of retinal reattachment following the scleral buckling procedure in infants with advanced ROP. On the contrary, we manipulated the data occurring from the visual outcome to facilitate the interpretation of the results. Therefore, we dichotomised the six categories of visual impairments, as proposed by the WHO, into two categories that demonstrate the success of moderate visual impairment as opposed to more severe visual impairment or blindness.

**Figure 2:**
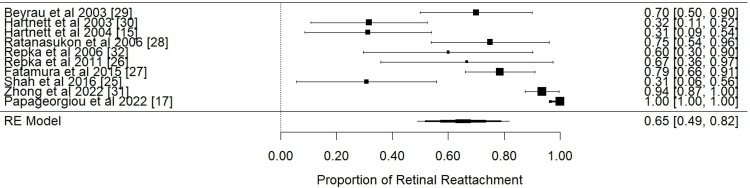
Anatomical outcome: forest plot of proportional meta-analysis for retinal reattachment

**Figure 3:**
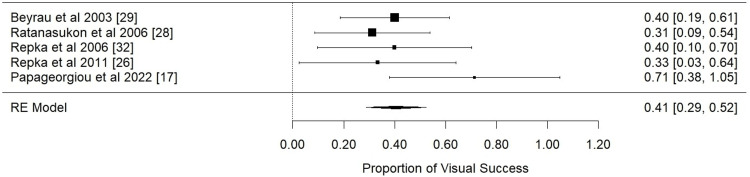
Visual outcome: forest plot of proportional meta-analysis for visual impairment

A summary of the individual proportions of eyes that succeeded retinal reattachment and moderate visual impairment is represented in Table 7. Additionally, subsequent meta-analysis of the anatomical and visual outcomes is demonstrated in forest plots in Figures 2, 3, respectively. 

**Table 7:** Proportional analysis of the included studies Anatomical outcome: proportion of retinal reattachment; visual outcome: proportion of favourable visual results, defined as normal visual acuity or mild visual impairment

Author and year	Anatomical outcome	Visual outcome
Beyrau et al. 2003 [29]	0.70	0.40
Hartnett et al. 2003 [30]	0.32	-
Hartnett et al. 2004 [15]	0.31	-
Ratanasukon et al. 2006 [28]	0.75	0.31
Repka et al. 2006 [32]	0.60	0.40
Repka et al. 2011 [26]	0.67	0.33
Fatamura et al. 2015 [27]	0.79	-
Shah et al. 2016 [25]	0.30	-
Zhong et al. 2022 [31]	1	-
Papageorgiou et al. 2022 [17]	0.94	0.71

Anatomical outcome: Data from all 10 studies were used for the analysis of the success of retinal reattachment following scleral buckle surgery in infants with advanced ROP. The pooled prevalence across all studies was calculated at 0.65 (95% Cl [0.49 - 0.82], p-value 0 < 0.0001). This means that of all eyes that sustained scleral buckle surgery, retinal reattachment was achieved in 65% of them. We also calculated the prediction intervals that estimate the true treatment effect that may be anticipated in future analyses in different settings. We used the Wilson-Score method due to the small sizes of individual studies. The prevalence of retinal reattachment was measured at 0.71 (71%) (95% Cl [0.6413; 0.7626]). The I2 was measured at 95.88%, which indicates considerable heterogeneity among the studies included in the meta-analysis. High heterogeneity is expected in proportional meta-analyses, considering the existence of variance and different setting characteristics in studies with small sizes. 

Visual outcome: The data derived from five studies were incorporated into the proportional single-arm meta-analysis regarding the visual impact of scleral buckling surgery. The pooled effect size estimate was calculated at 0.41 (95% Cl [0.29; 0.52], p-value < 0.0001, I2 =0.02%). Prevalence was 0.40 with a prediction interval of 95% Cl between 0.29 and 0.53, using the Wilson-Score method. No further sensitivity analysis was required as heterogeneity was insignificant. 

Discussion

In this systematic review and proportional single-arm meta-analysis, we assessed the anatomical and visual outcomes after scleral buckling surgery in infants with advanced ROP. We focused on the retinal reattachment and the vision impairment, as per WHO recommendations, occurring following the procedure. Our analysis included 10 studies and 214 eyes in total, providing an adequate sample of comprehensive material to interpret. The prevalence of retinal reattachment was calculated at 65% (95% Cl [0.49 - 0.82], p-value 0 < 0.0001), whereas the prevalence of normal VA or mild visual impairment was estimated at 41% (95% Cl [0.29; 0.52], p-value < 0.0001, I2 =0.02%).

Scleral buckling surgery in ROP is performed either as a standalone primary procedure or as an adjacent to vitrectomy surgical procedure. Especially prior to the development and the establishment of vitrectomy as a surgical technique, the scleral buckle had been the gold standard for the restoration of retinal detachments, occurring in advanced stages of ROP. Anatomic success, defined as the successful retinal reattachment following scleral buckle surgery in infants with ROP stage 4 or 5, was evaluated between 46% and 70%, in observational studies that were conducted prior to 2000 [16,34-37]. Ever since, a variety of studies, concentrating on the impact of scleral buckle, have been published.

Results obtained from studies conducted between 2000 and 2024 indicate that the mean proportion of retinal reattachment and successful anatomical outcomes ranges from 31.58% to 100%. Notably, it should be emphasized that less favourable results, approximately 30%, were observed in studies that included patients with ROP stage 5 [15,25,30]. On the contrary, the remaining studies that examined the impact of scleral buckling surgery on the anatomical success of the retina achieved a retinal reattachment rate exceeding 60% [17,26,27,28,29,31,32], with five of these studies surpassing 70% [17,27,28,29,31]. The majority of the population in these studies included infants with ROP stage 4A or 4B or patients with high-risk presthreshold disease (Type 1 ROP), as defined in the Early Treatment for Retinopathy of Prematurity Study (ETROP). Based on these findings, one could hypothesize that early intervention with scleral buckling in infants with ROP may enhance the success rate of the procedure and facilitate retinal reattachment. Another factor that warrants emphasis is the additional treatments administered to each population across various studies, including laser photocoagulation, cryotherapy, and intravitreal injections with anti-VEGF agents. However, these characteristics are not sufficiently delineated in the individual studies; consequently, further subgroup analysis has not been favoured.

Our results, occurring from the pooled meta-analysis of the 10 included studies, are consistent with the above results, indicating the efficiency of scleral buckling surgery in advanced stages of ROP. The overall anatomical success was evaluated at 65% in a total of 214 eyes. The heterogeneity among studies was notably high, at 95.88%. This result may potentially stem from the type of study conducted, as the prevalence and single-arm studies could influence the heterogeneity rate. Additionally, a variation in the timing of the surgical procedures may have contributed to the observed heterogeneity. Further subgroup analysis may not yield significant insights, as the sample size within individual studies is generally limited. Furthermore, the nature of the available data did not adequately support a more comprehensive analysis based on prior or additional treatments. Although the impact of supplementary treatments, specifically cryotherapy, laser photocoagulation, or anti-VEGF intravitreal therapy, has generally demonstrated favourable outcomes, particularly in infants diagnosed with ROP stage 4B, subsequent analysis should be performed in order to compare the effect of these treatments as either preoperative or concurrent therapeutic options. This observation would elucidate the efficacy of scleral buckle surgery in mitigating the progression of the disease and would support the rationale for a combined treatment approach that benefits patients. Such an approach may prove even more effective in individuals with high-risk prethreshold disease (Type 1 ROP). Furthermore, it is noteworthy that advancements in surgical equipment developed over the years have the potential to enhance the success rates of retinal reattachment following scleral buckling surgery.

The results regarding the visual outcome are variable and often disappointing. Beyrau et al. [29] suggested that, within a follow-up period of 3.5 to 28 months, the functional outcome of 12 eyes in which macula reattachment was achieved was favourable in eight eyes that presented fix and follow ability, while the rest of the eyes developed inability only to follow, inability to both fix and follow or optokinetic nystagmus. On the other hand, Ratanasukon et al. [28] reported the VA and cycloplegic refraction in patients that received scleral buckling surgery. Of the total of 16 eyes, only four eyes (50%) of stage 4A ROP and one eye (12.5%) of stage 4B ROP achieved favourable outcomes, which was defined as VA better than 20/200. The follow-up period was limited to a mean of 17.3 months, while the buckle was removed at 6.68 months (range 4.5 - 11 months) in 11 patients in whom retinal reattachment was achieved. Repka et al. [32], in 2006, measured the VA at nine months corrected age in 78 eyes that received either scleral buckle surgery or vitrectomy. The results were categorized as favourable (normal [> 3.70 cycles/degree] and below normal [> 1.85 to < 3.70 cycles/ degree]), poor (< 1.85 cycles/degree), low vision (detection of only the 2.2-cm-wide stripes of the Teller low vision card at any distance), light perception only, or no light perception. Overall, the visual outcome showed favourable results in 13 patients, of which four eyes had single scleral buckling, all in stage 4A. Likewise, Repka et al. [26], in 2011, similarly measured the VA through letter optotype testing in 89 eyes of 63 patients at 6 years of age. VA was categorized as normal (> 20/40), below normal (< 20/40 to > 20/200), poor (measurable acuity of < 20/200), or blind/low vision/light perception. Favourable acuity was defined as better than 20/200 and was measured in six eyes in total, of which 3 eyes received primary scleral buckling surgery. Interestingly, no eye with stage 5 ROP developed better vision than light perception. Finally, Papageorgiou et al. [17] presented the postoperative VA in seven eyes of five patients of stages 4A and 4B ROP, who underwent single scleral buckling surgery. Visual results are comparable with the anatomical outcomes as five eyes had only mild visual impairment.

It is interesting to note that according to our results, only 40% of the total population presented mild visual impairment or normal vision following scleral buckling surgery in advanced stages of ROP. The heterogeneity was low at 0.02%, although the result was not statistically significant (p-value for heterogeneity 0.39). This suggests that notable improvement in terms of functional and visual restoration is not guaranteed, even in patients with retinal reattachment after a scleral buckle in ROP stages 4A, 4B and 5. Preterm infants may experience numerous factors that contribute to visual impairment. The differences in the timing and the duration of follow-up time could be a potential cause. This might, also, be related to the anatomic and functional maturity of infants that are tested in each individual study and which can be present up until the 42 weeks of gestation [28]. Another factor that should be considered is the variety of methods and tests used for the assessment of VA in infants. Fixation behaviour, as well as grating VA systems, can overestimate the functional results and be significantly unreliable [26]. Moreover, the presence of subretinal fluid on a background of retinal detachment could affect VA or generate amblyopia in infants with advanced stages of ROP. Finally, scleral buckling surgery is strongly linked to refractive complications, specifically myopia. A refractive error should be calculated and corrected following the removal of the scleral buckle.

Both scleral buckling surgery and vitrectomy have several disadvantages. Scleral buckling presents intraoperative complications, primarily associated with the risk of scleral perforation, considering the small thickness of an infant’s sclera in comparison to that of an adult patient [34]. It is important to avoid a tight pulling of the scleral band as this can induce an increase in the intraocular pressure and subsequent disturbance in the retinal and choroidal circulation [38]. Another significant drawback is the induction of myopia and anisometropia, which can reach up to 12 dioptres, thereby leading to an increased risk for amblyopia [16]. Axial elongation and forward displacement of the lens contribute to both axial and lenticular high myopia, which predisposes individuals to amblyopia [17]. In fact, a case series study [35], conducted in 1996 involving 10 patients who underwent scleral buckling for stage 4A ROP, indicated the provocation of myopia, ranging from -5 dioptres to -15 dioptres, in all participants with a mean follow-up of 10.5 weeks. Consequently, a secondary surgical procedure is typically performed, for the removal of the buckle, aimed at mitigating or even preventing refractive errors and promoting the natural growth of the globe [34,39]. Encircling buckling, as opposed to segmental buckling, is also favoured for analogous reasons. Lastly, prudence should be exercised in instances of subretinal fluid drainage, as it is associated with subretinal haemorrhage and infection [40-42].

On the other hand, the complications associated with vitrectomy, either lens sparing vitrectomy (LSV) or lensectomy-vitrectomy (LV), are similarly concerning. Vitrectomy has been associated with intraoperative bleeding, iatrogenic retinal breaks, intraoperative retinal tears and corneal clouding, as has been reported in several studies [40,43]. Specifically, the formation of lens opacity and cataracts was calculated at 2.1% after LSV surgery in infants with stage 4A ROP, although the percentage of retinal reattachment was 82.1% (230/280). The rate of lensectomy following LSV was 12.5% [15,44]. Additionally, glaucoma has been previously documented as a potential complication of LV or LSV, which was managed either conservatively with topical antiglaucoma medications or surgically with filtering surgeries [31,45]. It is important to note that the preservation of an infant’s natural lens reduces the risk of glaucoma and facilitates vision development [16].

It is evident that both scleral buckling and vitrectomy should be adjusted to meet the specific needs of the patient. Research has demonstrated that primary scleral buckling is predominantly selected in cases of peripheral detachments when addressing the tractional forces proving challenging without lens removal, especially in infants diagnosed with stage 4A ROP [16]. Furthermore, it has been substantiated through fluorescence angiography that scleral buckling can stabilize the neovascular activity of fibrovascular tissue, downregulate the VEGF and reduce the progress of ROP [40]. Notably, when scleral buckle surgery is performed with concurrent anti-VEGF or laser photocoagulation treatment, the likelihood of success is significantly enhanced [25,27]. Other factors that should be considered include the necessity for specialised equipment and the presence of a trained vitreoretinal surgeon for the execution of LV or LSV, as well as the availability of advanced facilities equipped with NICUs to accommodate patients following the completion of each surgical procedure [17]. Finally, a comparative analysis of the costs associated with scleral buckling and pars plana vitrectomy (PPV) for adult retinal detachments revealed that when accounting for all expenses, including potential cataract surgery, scleral buckling procedures were found to be 10.7% less expensive than PPV for retinal detachment repair in phakic patients [46]. In conclusion, it is advisable for ophthalmic surgeons to select the appropriate surgical technique based on the anatomical status of the patients, their comorbidities, and the available equipment.

Limitations

To the best of our knowledge, this represents the sole meta-analysis investigating the anatomic and functional outcomes of scleral buckling surgery in infants with advanced ROP. A systematic review published in 2004 encompassed eight studies conducted between 1994 and 2001, which compared scleral buckling to vitrectomy for ROP stages 4 and 5, with respect to the functional and visual success of these procedures [38]. However, our study has several limitations. Firstly, the included studies are not randomised. Observational studies, such as case series or cohort studies with limited populations, lack the methodological power of nonrandomised controlled trials and may yield biased results. Also, the existing literature is deficient in large prospective studies with extensive follow-up periods that would provide more reliable outcomes. Furthermore, the variability in the reported follow-up periods may significantly influence the outcomes. Thus, it is recommended that future studies incorporate extended follow-up durations. Finally, the examination of small eyes in premature infants, as well as the ambiguity in differentiating stage 4A from stage 4B ROP disease, present inherent challenges, and any inaccuracies in preoperative and postoperative staging, especially with respect to the measurement of the VA, should be duly acknowledged.

## Conclusions

In summary, our systematic review and meta-analysis elucidated the role of scleral buckling surgery in the management of ROP in stages 4 and 5 among premature infants. The 10 studies included in this analysis demonstrated that the anatomical outcomes were more favourable compared to the visual outcomes. The efficacy of scleral buckling surgery is unequivocal, particularly in the early advanced stages of ROP and in conjunction with combination therapies. Nevertheless, it is recommended that future prospective studies be conducted with extended follow-up periods and larger populations to further enhance the existing body of knowledge.
